# Prognostic Value of *KRAS*/*TP53* Status for Overall Survival in First-Line Monoimmunotherapy and Chemoimmunotherapy Treated Patients With Nonsquamous NSCLC in the Netherlands: A Brief Report

**DOI:** 10.1016/j.jtocrr.2024.100745

**Published:** 2024-10-17

**Authors:** Vincent D. de Jager, Léon C. van Kempen, Betzabel N. Cajiao Garcia, T. Jeroen N. Hiltermann, Anthonie J. van der Wekken, Ed Schuuring, Stefan M. Willems

**Affiliations:** a Department of Pathology and Medical Biology, University of Groningen, University Medical Center Groningen, Groningen, The Netherlands; b Department of Pathology, University Hospital Antwerp, University of Antwerp, Antwerp, Belgium; c Department of Pulmonary Diseases and Tuberculosis, University of Groningen, University Medical Center Groningen, Groningen, The Netherlands

**Keywords:** NSCLC, *KRAS*, *TP53*, Immunotherapy, Chemoimmunotherapy, Real-world data

## Abstract

**Introduction:**

Programmed death-ligand 1 (PD-L1) is the main predictive biomarker used to identify patients with NSCLC who are eligible for treatment with immune checkpoint inhibitors. Despite its utility, the predictive capacity of PD-L1 is limited, necessitating the exploration of supplementary predictive biomarkers. In this report, we describe the prognostic value of *KRAS*/*TP53* mutation status for overall survival (OS) in patients with NSCLC treated with first-line immunotherapy or combined chemoimmunotherapy.

**Methods:**

Clinical data of all patients diagnosed with metastatic nonsquamous NSCLC in the Netherlands between January 1 and December 31 of 2019 were retrieved from the Netherlands Cancer Registry and linked to pathology reports of the Dutch Nationwide Pathology Databank. A total of 694 patients with available *KRAS* and *TP53* mutation status and treated with first-line pembrolizumab or chemoimmunotherapy were included, with a median follow-up time of 42.5 months. Patients with an *EGFR* or *MET* mutation or *ALK*, *ROS1,* or *RET* fusion were excluded from the analysis.

**Results:**

Among patients treated with first-line pembrolizumab or chemoimmunotherapy, mutations in *KRAS* and *TP53* occurred in 48.8% (n = 339) and 58.4% (n = 405), respectively. OS differed significantly between *KRAS*/*TP53* mutational subgroups in patients treated with first-line pembrolizumab or chemoimmunotherapy (log-rank test, *p* = 0.007). Median OS of pembrolizumab or chemoimmunotherapy treated patients with mutated *TP53* was longer in patients with *KRAS*-wildtype (485 versus 359 d, hazard ratio [HR] = 0.76, *p* = 0.028) or mutated *KRAS* (571 versus 447 d, HR = 0.73, *p* = 0.019). In a separate analysis of treatment subgroups, mutated *TP53* was associated with longer median OS in chemoimmunotherapy treated *KRAS*-wildtype patients (468 versus 341 d, HR = 0.71, *p* = 0.029) but not in monoimmunotherapy treated patients with *KRAS*-wildtype (512 versus 371 d, HR = 0.91, *p* = 0.78). In multivariable Cox regression analysis including age, sex, clinical disease stage, and PD-L1 tumor proportion score, *KRAS*/*TP53* mutation status was no longer associated with OS.

**Conclusions:**

Among patients with metastatic NSCLC who are treated with pembrolizumab or chemoimmunotherapy, the presence of a pathogenic *TP53* and *KRAS* mutation is associated with longer OS. Nevertheless, in multivariable Cox regression analysis including age, sex, clinical disease stage, and PD-L1 tumor proportion score, *KRAS*/*TP53* mutation status was no longer associated with OS.

## Introduction

Programmed death-ligand 1 (PD-L1) immunohistochemistry is the main predictive tumor marker used to identify patients with advanced-stage NSCLC who are eligible for first-line treatment with immune checkpoint inhibitors. Despite its utility, the predictive capacity of PD-L1 is restricted, necessitating the exploration of supplementary predictive biomarkers. In a recent report by Bischoff et al.,^1^ the authors reported the predictive and prognostic significance of *KRAS* and *TP53* mutations in determining the efficacy of first-line pembrolizumab treatment among a cohort of German patients with nonsquamous PD-L1-high (≥50%) NSCLC. Their findings underscored that within this patient subset, the concurrent presence of both a *KRAS* G12C and *TP53* mutation was associated with increased response rates to first-line mono-pembrolizumab treatment, accompanied by prolonged progression-free survival and overall survival (OS). Notably, a study by Tønnesen et al.^2^ corroborated these survival trends among patients treated with monoimmunotherapy or combined chemoimmunotherapy. Nevertheless, this study was constrained by a modest cohort size and limited follow-up duration.^2^

In the Netherlands, comprehensive real-world data pertaining to cancer patients, including pathology reports, initial treatment regimens, and OS outcomes, are systematically recorded in national databases.^3^^,^^4^ Using an existing real-world data set of patients diagnosed with NSCLC in 2019 in the Netherlands,^5^ we examined whether differences in survival pattern on the basis of *KRAS*/*TP53* mutational status could be observed in the entire population of patients with metastatic NSCLC who were treated with first-line immunotherapy, either as monotherapy or in combination with chemotherapy.

## Materials and Methods

As described previously, clinical data and pathology reports of patients diagnosed with metastatic nonsquamous NSCLC (pulmonary adenocarcinoma, NSCLC–not otherwise specified, and pulmonary adenosquamous carcinoma) in the Netherlands between January 1 and December 31 of 2019 were retrieved from the National Cancer Registry managed by the Netherlands Comprehensive Cancer Organization and the national pathology database (Palga), respectively.^5^ Histopathological diagnosis, PD-L1 tumor proportion score (TPS), and mutation status of *KRAS* and *TP53* were manually collected from the pathology reports. Molecular testing data were based on the reported results of routine biomarker testing, as performed at the discretion of the requesting individual and the testing laboratory. If more than one PD-L1 staining was performed, the highest PD-L1 TPS was included. Patients with an aberration of *EGFR*, *ALK,* or *RET* were excluded from the analysis. Classification of *TP53* variants was on the basis of the UMD TP53 database (accessed on June 1, 2024).^6^ Only pathogenic, likely pathogenic, and possibly pathogenic *TP53* variants were included as mutated *TP53* (*TP53*mut). This study was approved by the privacy and scientific committee of Palga and the ethical committee of the Netherlands Comprehensive Cancer Organization (K23.108). Informed consent was not required due to the study design. Cox regression analysis and log-rank tests were performed to obtain hazard ratios (HRs) and determine differences in OS between subgroups. For demographics of mutational subgroups, categorical and continuous variables were compared with the Pearson chi-square test and Kruskal-Wallis test, respectively. Statistical analyses were performed using IBM SPSS Statistics for Windows, version 28.0.0.0 (SPSS Inc., Chicago, IL). Figures were created using GraphPad Prism version 9.1.0. for Windows (GraphPad Software, San Diego, CA).

## Results

### Patient Characteristics

Patient characteristics are presented in Table 1. The median age at diagnosis was 65 years and 51% of patients were male individuals. Histopathological diagnosis was predominantly adenocarcinoma (90%), followed by NSCLC–not otherwise specified (9%).

**Table 1:** Patient Characteristics

Variables	Entire cohort (N = 694)	*KRAS*wt/*TP53*wt (n = 90)	*KRAS*wt/*TP53*mut (n = 265)	*KRAS*mut/*TP53*wt (n = 199)	*KRAS*mut/*TP53*mut (n = 140)	*p* Value
Age in y, median (IQR)	65 (58–71)	67 (60–72)	66 (59–72)	65 (58–71)	62 (57–69)	**0.01**
Male sex	356 (51)	49 (54)	153 (58)	92 (46)	62 (44)	**0.02**
Histology						0.77
Adenocarcinoma	625 (90)	81 (90)	236 (89)	177 (89)	131 (94)	
NSCLC-NOS	64 (9)	8 (9)	27 (10)	20 (10)	9 (6)	
Adenosquamous carcinoma	5 (1)	1 (1)	2 (1)	2 (1)	0 (0)	
Clinical stage of metastatic disease						0.11
M1a	130 (19)	20 (22)	42 (16)	42 (21)	26 (19)	
M1b	105 (15)	10 (11)	50 (19)	20 (10)	25 (18)	
M1c	459 (66)	60 (67)	173 (65)	137 (69)	89 (64)	
PD-L1 tested						0.13
Yes	682 (98)	87 (97)	263 (99)	193 (97)	139 (99)	
No	12 (2)	3 (3)	2 (1)	6 (3)	1 (1)	
PD-L1 score (if tested)						**<0.01**
<1%	234 (34)	53 (61)	88 (33)	72 (37)	21 (15)	
1%–49%	151 (22)	12 (14)	61 (23)	45 (23)	33 (24)	
50%–100%	292 (43)	22 (25)	112 (43)	75 (39)	83 (60)	
Inconclusive	4 (1)	0 (0)	2 (1)	1 (1)	1 (1)	
Unknown	1 (0)	0 (0)	0 (0)	0 (0)	1 (1)	
Treatment						**<0.01**
Pembrolizumab	237 (34)	18 (20)	93 (35)	63 (32)	63 (32)	
Chemoimmunotherapy	457 (66)	72 (80)	172 (65)	136 (68)	77 (68)	

*Note:* Categorical data are presented as n (%) unless otherwise specified. Boldface values indicate statistical significance (*p* value < 0.05).

IQR, interquartile range; wt, wildtype; mut, mutated; NSCLC-NOS, NSCLC–not otherwise specified; PD-L1, programmed death-ligand 1.

Among patients treated with pembrolizumab or chemoimmunotherapy, mutations in *KRAS* and *TP53* occurred in 48.8% (n = 339) and 58.4% (n = 405), respectively. Differences in age at diagnosis, sex, PD-L1 TPS, and the first line of treatment were observed between *KRAS* and *TP53* mutational subgroups.

### Survival Analysis of Mutational Subgroups

**Figure 1:**
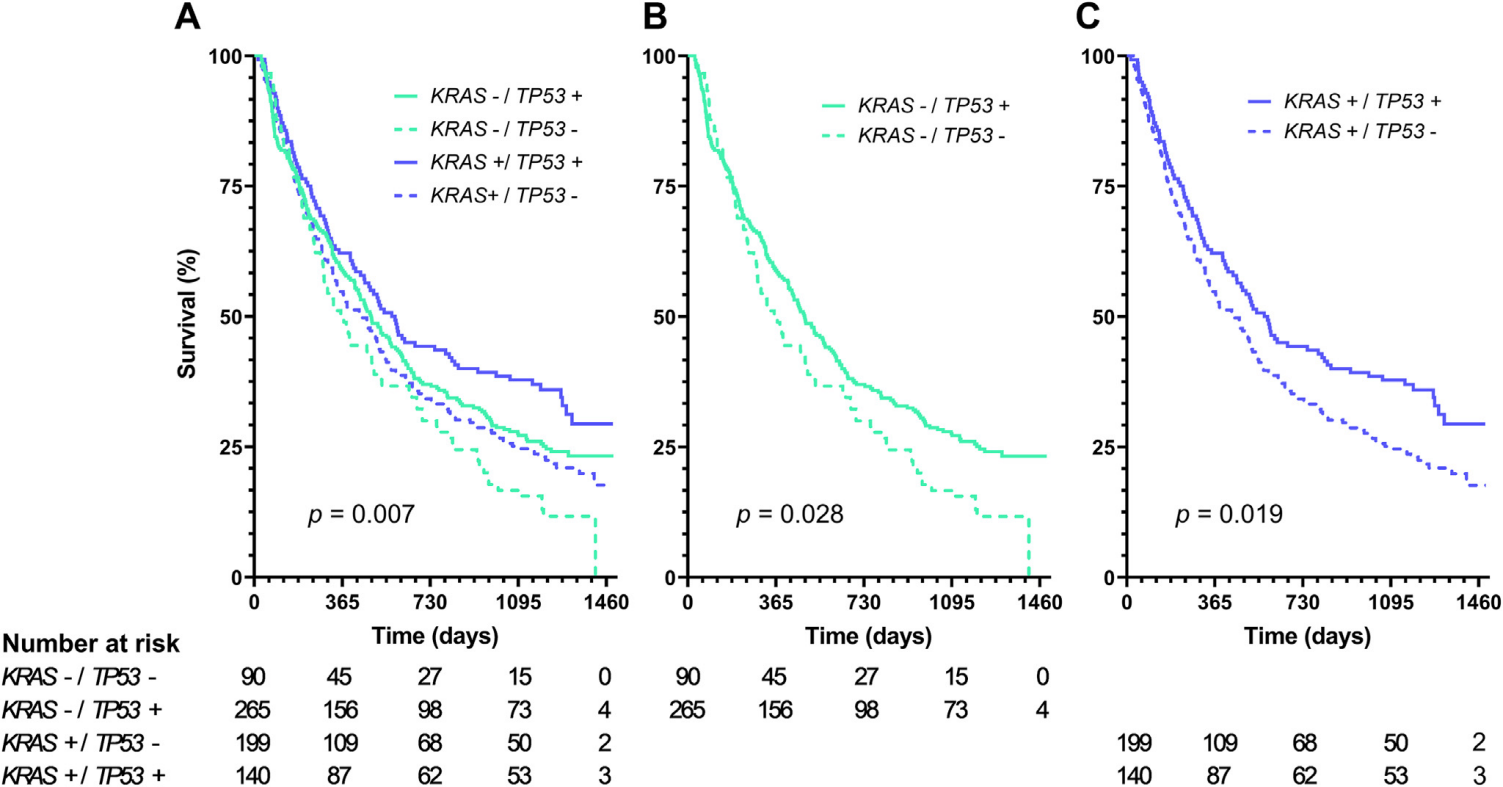
Overall survival of metastatic patients with NSCLC treated with first-line pembrolizumab or chemoimmunotherapy, stratified by *KRAS* and *TP53* mutational status: (*A*) all mutational subgroups, (*B*) *KRAS*-wildtype only, (*C*) mutated *KRAS* only. The presence of a mutation in respective genes is indicated by ‘+’ and the absence of a (likely) pathogenic mutation is indicated by ‘−’. Data comprised patients diagnosed in 2019 and were retrieved from the Dutch NCR and the Dutch National Pathology Database (Palga). NCR, National Cancer Registry.

The median follow-up time of the entire study population was 42.5 months. OS differed significantly between *KRAS*/*TP53* mutational subgroups in patients treated with first-line pembrolizumab or chemoimmunotherapy (log-rank test, *p* = 0.007) (Fig. 1*A*). Median OS of pembrolizumab or chemoimmunotherapy treated patients with *TP53*mut was longer among patients with *KRAS*-wildtype (*KRAS*wt) (485 versus 359 d, HR = 0.76, *p* = 0.028; Fig. 1*B*) and mutated *KRAS* (*KRAS*mut) (571 versus 447 d, HR = 0.73, *p* = 0.019; Fig. 1*C*). Further subclassification of patients with *KRAS*mut into G12C and non-G12C mutations revealed similar survival patterns, but differences between survival curves of mutated and nonmutated *TP53* no longer reached statistical significance (Supplementary Fig. 1).

Separate mutational subgroup survival analyses for pembrolizumab-treated patients and chemoimmunotherapy-treated patients are presented in Supplementary Figures 2 and 3. The presence of a *TP53* mutation was associated with longer OS in combination with *KRAS*wt in patients treated with first-line chemoimmunotherapy (Supplementary Fig. 3), but not in patients with *KRAS*mut NSCLC (Supplementary Figure 2).

Univariable and multivariable Cox regression analyses were performed using age, sex, clinical stage of metastatic disease, *KRAS*/*TP53* mutation status, and treatment category as independent variables (Supplementary Table 1). In univariable Cox regression analysis, higher age, chemoimmunotherapy treatment (compared with monoimmunotherapy), higher stage of clinical stage metastatic disease (compared with cM1a), PD-L1 TPS less than 1% (compared with PD-L1 TPS ≥ 50%), and *KRAS*wt/*TP53*wt or *KRAS*mut/*TP53*wt status (compared with *KRAS*mut/*TP53*mut) were associated with shorter OS. In multivariable Cox regression analysis, *KRAS*/*TP53* mutation status was no longer significantly associated with shorter OS, unlike other included independent variables (age, clinical stage of metastatic disease, and PD-L1 TPS).

## Discussion

Expression of PD-L1 is the main predictive biomarker for immunotherapy treatment. Nevertheless, as many patients do not respond to monoimmunotherapy or chemoimmunotherapy despite high or any PD-L1 expression, respectively, its utility is limited. In this study, we describe the prognostic value of *KRAS*/*TP53* mutational status in patients with metastatic NSCLC treated with first-line pembrolizumab or combined chemoimmunotherapy. In short, in our study population, *TP53*mut was associated with longer OS in both patients with *KRAS*wt and *KRAS*mut (Fig. 1). For *KRAS*mut, this was predominantly because of the survival pattern of first-line patients treated with pembrolizumab (Supplementary Fig. 2), while for *KRAS*wt, it was due to the survival pattern of combined chemoimmunotherapy treated patients (Supplementary Fig. 3). Nevertheless, in multivariable Cox regression analysis involving age, sex, clinical disease stage, and PD-L1 TPS, *KRAS*/*TP53* mutation status was no longer associated with OS. Overall, these real-world data indicate the potential prognostic significance of combined *KRAS*/*TP53* mutational status in a more heterogeneous patient population compared with the patient population reported by Bischoff et al.,^1^ and in a larger patient group than presented by Tønnesen et al.^2^

In addition, in nonimmunotherapy treated patients with NSCLC, the mutational status of *TP53* may be relevant. For example, the occurrence of a somatic *TP53* mutation has been associated with lower response rates and shorter progression-free survival and OS in patients with NSCLC receiving targeted therapy for an *EGFR* mutation or *ALK* fusion.^7^^,^^8^ For patients with *EGFR*-mutated NSCLC, a post hoc analysis of the RELAY (Ramucirumab Plus Erlotinib Versus Placebo) trial indicated that the addition of ramucirumab (a vascular endothelial growth factor receptor 2 inhibitor) to erlotinib (a first-generation EGFR tyrosine kinase inhibitor) improves treatment outcome in patients with a concurrent *TP53* mutation.^9^ Thus, as a prognostic and predictive biomarker, it may be relevant to perform *TP53* mutation testing in patients with *EGFR*-mutated NSCLC. By contrast, in patients eligible for first-line monoimmunotherapy, *TP53*mut has been associated with better treatment outcomes when co-occurring with a *KRAS* G12C mutation^1^^,^^10^^,^^11^ One retrospective study also described this association in patients treated with immunotherapy in second- or later-line of therapy.^12^ Importantly, the prognostic effect of *TP53*mut was not described in patients with *KRAS*wt NSCLC. Although some of these studies included patients from multiple centers, none included patients treated with first-line combined chemoimmunotherapy. In addition, previous real-world data studies that included patients treated with first-line combined chemoimmunotherapy have been limited by the modest size of patient cohorts.^2^^,^^13^^,^^14^ Our current study confirms the potential value of combined *KRAS*/*TP53* mutational status as a prognostic biomarker in immunotherapy-treated patients and adds the prognostic value of *TP53*mut for combined chemoimmunotherapy in patients with *KRAS*wt NSCLC.

A potential explanation for why patients with *TP53*mut NSCLC have better clinical outcomes on immunotherapy or chemoimmunotherapy treatment is that the loss of the tumor suppressive function of p53 may lead to higher mutation rates and increased numbers of tumor neo-antigens in patients with *TP53*mut NSCLC.^13^ As a result, it has been suggested that *TP53*mut may serve as a surrogate marker for tumor mutational burden.^13^

Similar to the aforementioned RELAY trial, a post hoc analysis of the IMpower150 trial indicated that the addition of bevacizumab (a vascular endothelial growth factor A inhibitor) and atezolizumab to carboplatin/paclitaxel chemotherapy improves treatment outcomes in patients with *KRAS*mut NSCLC with concurrent *TP53* mutation compared with combining atezolizumab and chemotherapy only.^15^ Thus, in patients with *KRAS*mut receiving chemoimmunotherapy, *TP53*mut may be a predictive biomarker for antiangiogenic therapy.

This study has several limitations. First, the use of real-world data from routine molecular testing may lead to incomplete detection of mutations and selection bias. For example, some laboratories may only include testing to detect hotspot *TP53* mutations, leading to pathogenic non-hotspot mutations remaining undetected. In addition, some laboratories may not have routinely tested *TP53*, thereby resulting in these patients not being included in this study, causing selection bias of only patients tested in other laboratories. Second, the design of a nationwide single-year cohort of the Netherlands may limit the generalizability of the findings to other geographic regions and patient populations of more recent years with potential changes in treatment protocols.

With emerging evidence for the prognostic and potential predictive value of immunotherapy, chemoimmunotherapy, and antiangiogenic therapy, the addition of *TP53* mutation testing in patients with *KRAS*mut NSCLC may be warranted. Unlike *KRAS*, the mutational status of *TP53* is currently not incorporated into standard decision-making guidelines for NSCLC. Nevertheless, the combined use of these biomarkers holds promise for refining patient selection considerations in the context of first-line immunotherapy or chemoimmunotherapy treatment.

## CRediT Authorship Contribution Statement

**Vincent D. de Jager:** Conceptualization, Methodology, Formal analysis, Investigation, Data curation, Writing - original draft, Writing - review & editing, Visualization.

**Léon C. van Kempen:** Conceptualization, Methodology, Investigation, Writing - review & editing.

**Betzabel N. Cajiao Garcia:** Data curation, Methodology, Investigation, Writing - review & editing.

**T. Jeroen N. Hiltermann:** Methodology, Investigation, Writing - review & editing.

**Anthonie J. van der Wekken:** Conceptualization, Methodology, Investigation, Writing - review & editing.

**Ed Schuuring:** Conceptualization, Methodology, Investigation, Writing - review & editing, Supervision.

**Stefan M. Willems**: Conceptualization, Methodology, Investigation, Writing - review & editing, Supervision.

## Disclosure

Dr. de Jager has received speaker’s fees from Roche and Janssen (Johnson&Johnson) (all paid to the institution). Dr. van Kempen has received grants or contracts from 10.13039/100002429Amgen, 10.13039/100004325AstraZeneca, 10.13039/100004326Bayer, Janssen-Cilag, Merck, Roche, and Servier (all payments to institution), has received payment or honoraria for lectures, presentations, speakers bureaus, manuscript writing, or educational events from Amgen, AstraZeneca, Bayer, Bristol-Myers Squibb, Eli Lilly, Novartis, Pfizer, and Roche (all payments to institution), has received support for attending meetings or travel from Roche (payments to institution), has participated on a Data Safety Monitoring Board or Advisory Board for Janssen-Cilag, Merck, and Roche (all payments to institution), has a leadership or fiduciary role in Commission Personalized Medicine – Belgium (unpaid), has stock or stock options in Cyclomis (institutional/personal). Dr. Hiltermann has received grants or contracts from 10.13039/100004337Roche, AstraZeneca, and Bristol-Myers Squibb, and has leadership or fiduciary roles in CieBOM. Dr. Wekken has received grants or contracts from AstraZeneca, Boehringer Ingelheim, Pfizer, Roche, and Takeda, has received consulting fees from AstraZeneca, Janssen, Eli Lilly, Roche, and Takeda, has received payment or honoraria for lectures, presentations, speakers bureaus, manuscript writing or educational events from AstraZeneca, Bristol-Myers Squibb, Eli Lilly, Pfizer, and Roche, has a leadership or fiduciary role in oncology section NVALT, guideline committee NSCLC and CUP, dure geneesmiddelen committee NVALT and FMS. Dr. Schuuring has received grants or contracts from Abbott, Biocartis, AstraZeneca, Invitae/Archer, Bayer, Bio-Rad, Roche, Agena Bioscience, CC Diagnostics, Merck Sharp & Dohme/Merck, and SNN/EFRO (all (unrestricted) grants paid to UMCG institution), has received consulting fees from Merck Sharp & Dohme/Merck, AstraZeneca, Roche, Novartis, Bayer, Bristol-Myers Squibb, Eli Lilly, Amgen, Agena Bioscience, CC Diagnostics, and Janssen-Cilag (Johnson&Johnson) (all advisory board (incidental) and travel expenses (honoria/grant paid to UMCG institution)), and Astellas Pharma, GlaxoSmithKline, Sinnovisionlab, Sysmex, and Protyon (all advisory board (incidental) (honoria/grant paid to UMCG institution)), has received payments or honoraria for lectures, presentations, speakers bureaus, manuscript or educational events from Bio-Rad, Seracare, Roche, Biocartis, Eli Lilly, Agena Bioscience, Illumina, has received support for attending meeting or travel from Bio-Rad, Biocartis, Ageno Sciences, Illumina, Roche/Foundation Medicine, and QCMD, is board member of Dutch Society of Pathology (unpaid), European Society of Pathology (unpaid), European Liquid Biopsy Society (unpaid), is secretary/member of advisory committee for assessment of molecular diagnostics (cieBOD) (honoraria paid to UMCG institution), is member of the national guideline advisory committee (honoraria paid to UMCG institution). Dr. Willems has received grants or contracts from Roche, Bayer, Eli Lilly, Pfizer, AstraZeneca, Merck Sharp & Dohme, Amgen, and Novartis (unrestricted research grants), and has a leadership or fiduciary role in the strategic advisory board of Roche. Dr. Garcia declares no conflict of interest. The authors thank the registration team of the Netherlands Comprehensive Cancer Organisation (IKNL) for the collection of data for the Netherlands Cancer Registry as well as IKNL scientific staff for scientific advise.

## References

[1] Bischoff P., Reck M., Overbeck T. (2024). Outcome of first-line treatment with pembrolizumab according to KRAS/TP53 mutational status for nonsquamous programmed death-ligand 1-high (>/=50%) NSCLC in the German national network genomic medicine lung cancer. J Thorac Oncol.

[2] Tønnesen E.M.T., Stougaard M., Meldgaard P., Lade-Keller J. (2023). Prognostic value of KRAS mutations, TP53 mutations and PD-L1 expression among lung adenocarcinomas treated with immunotherapy. J Clin Pathol.

[3] Garcia B.N.C., van Kempen L.C., Kuijpers C.C.H.J., Schuuring E., Willems S.M., van der Wekken A.J. (2022). Prevalence of KRAS p.(G12C) in stage IV NSCLC patients in the Netherlands; a nation-wide retrospective cohort study. Lung Cancer.

[4] Gijtenbeek R.G.P., Damhuis R.A.M., van der Wekken A.J., Hendriks L.E.L., Groen H.J.M., van Geffen W.H. (2023). Overall survival in advanced epidermal growth factor receptor mutated non-small cell lung cancer using different tyrosine kinase inhibitors in the Netherlands: a retrospective, nationwide registry study. Lancet Reg Health Eur.

[5] de Jager V.D., Cajiao Garcia B.N., Kuijpers C.C.H.J. (2024). Regional differences in predictive biomarker testing rates for patients with metastatic NSCLC in the Netherlands. Eur J Cancer.

[6] Hamroun D., Kato S., Ishioka C., Claustres M., Béroud C., Soussi T. (2006). The UMD TP53 database and website: update and revisions. Hum Mutat.

[7] Stockhammer P., Grant M., Wurtz A. (2024). Co-occurring alterations in multiple tumor suppressor genes are associated with worse outcomes in patients with EGFR-mutant lung cancer. J Thorac Oncol.

[8] Qin K., Hou H., Liang Y., Zhang X. (2020). Prognostic value of TP53 concurrent mutations for EGFR- TKIs and ALK-TKIs based targeted therapy in advanced non-small cell lung cancer: a meta-analysis. BMC Cancer.

[9] Nishio M., Paz-Ares L., Reck M. (2023). RELAY, ramucirumab plus erlotinib (RAM+ERL) in untreated metastatic EGFR-mutant NSCLC (EGFR+ NSCLC): association between TP53 status and clinical outcome. Clin Lung Cancer.

[10] Frost N., Kollmeier J., Vollbrecht C. (2021). KRAS^G12C^/TP53 co-mutations identify long-term responders to first line palliative treatment with pembrolizumab monotherapy in PD-L1 high (≥50%) lung adenocarcinoma. Transl Lung Cancer Res.

[11] Wang S., Jiang M., Yang Z., Huang X., Li N. (2022). The role of distinct co-mutation patterns with TP53 mutation in immunotherapy for NSCLC. Genes Dis.

[12] Liu J., Gao J. (2023). Efficacy of immunotherapy as second-line or later-line therapy and prognostic significance of KRAS or TP53 mutations in advanced non-small cell lung cancer patients. Eur J Cancer Prev.

[13] Assoun S., Theou-Anton N., Nguenang M. (2019). Association of TP53 mutations with response and longer survival under immune checkpoint inhibitors in advanced non-small-cell lung cancer. Lung Cancer.

[14] Mathiot L., Nigen B., Goronflot T. (2024). Prognostic impact of TP53 mutations in metastatic nonsquamous non-small-cell lung cancer. Clin Lung Cancer.

[15] West H.J., McCleland M., Cappuzzo F. (2022). Clinical efficacy of atezolizumab plus bevacizumab and chemotherapy in KRAS-mutated non-small cell lung cancer with STK11, KEAP1, or TP53 comutations: subgroup results from the phase III IMpower150 trial. J Immunother Cancer.

