# Supplementary material for: Prognostic Value of KRAS/TP53 Status for Overall Survival in First-Line Monoimmunotherapy and Chemoimmunotherapy Treated Patients With Nonsquamous NSCLC in the Netherlands: A Brief Report
Source: JTO Clin Res Rep. 2024 Oct 17;5(12):100745. doi: 10.1016/j.jtocrr.2024.100745 (PMC11671682; doi:10.1016/j.jtocrr.2024.100745)
Supplement: Supplementary Figures 1-3 and Supplementary Table 1 [file mmc1.docx]

**Supplementary Figure S1.**


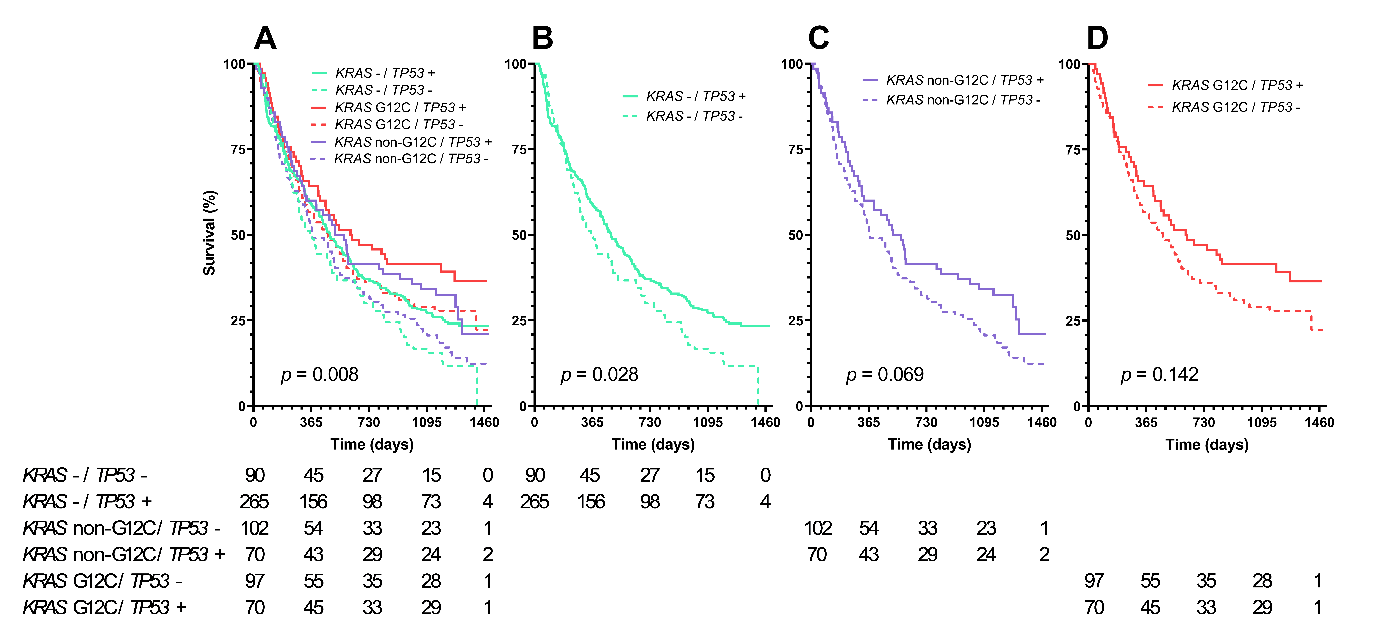


**Supplementary Figure S1**. Overall survival of metastatic NSCLC patients treated with first-line pembrolizumab or chemo-immunotherapy, stratified by *KRAS* and *TP53* mutational status: (A) all mutational subgroups, (B) *KRAS* wildtype only, (C) *KRAS* non-G12C only, and (D) *KRAS* G12C only. Presence of mutation in respective genes is indicated by ‘+’, lack of a (likely) pathogenic mutation is indicated by ‘-’. Data comprise patients diagnosed in 2019 and were retrieved from the Dutch National Cancer Registry (NCR) and the Dutch national pathology database (Palga).

**Supplementary Figure S2.**

**
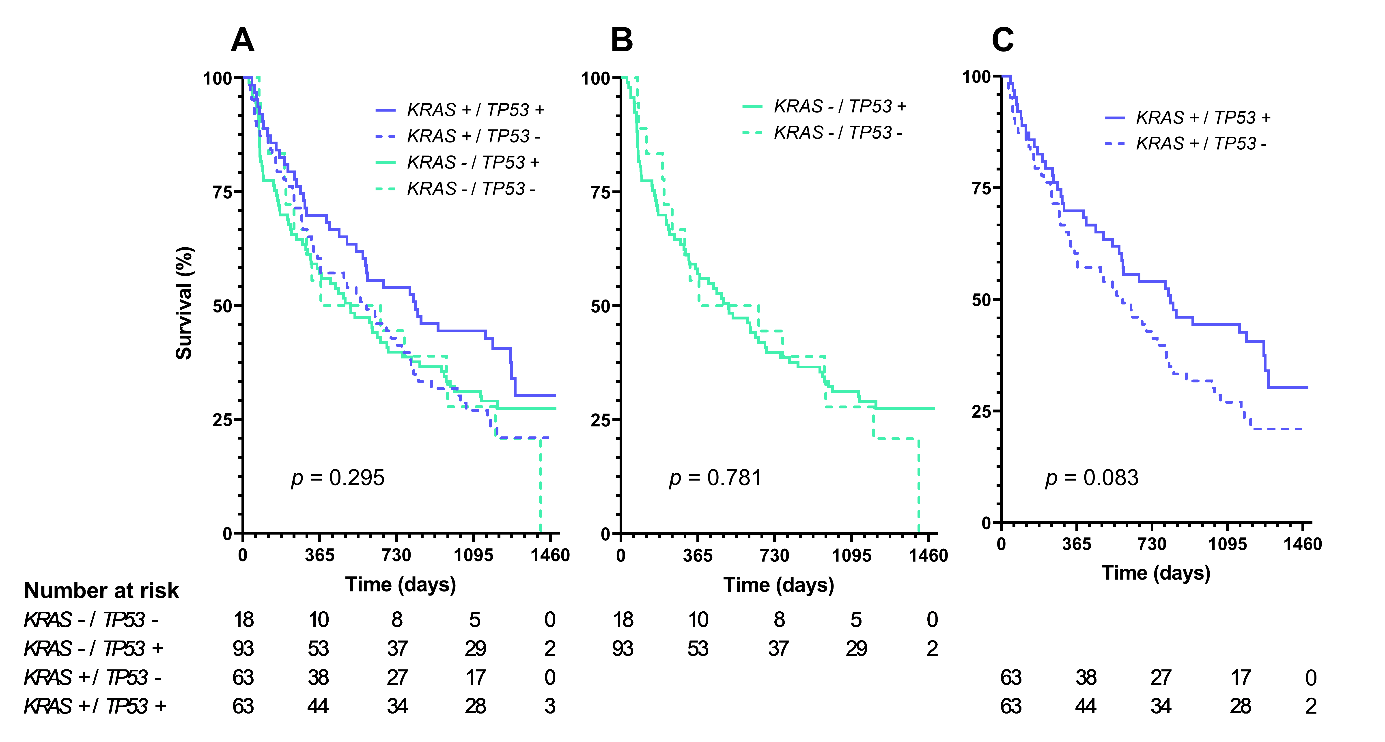
**

**Supplementary Figure S2**. Overall survival of metastatic NSCLC patients treated with first-line pembrolizumab, stratified by *KRAS* and *TP53* mutational status: (A) all mutational subgroups, (B) *KRAS* wildtype only, and (C) mutated *KRAS* only. Presence of mutation in respective genes is indicated by ‘+’, lack of a (likely) pathogenic mutation is indicated by ‘-’. Data comprise patients diagnosed in 2019 and were retrieved from the Dutch National Cancer Registry (NCR) and the Dutch national pathology database (Palga).

**Supplementary Figure S3.**


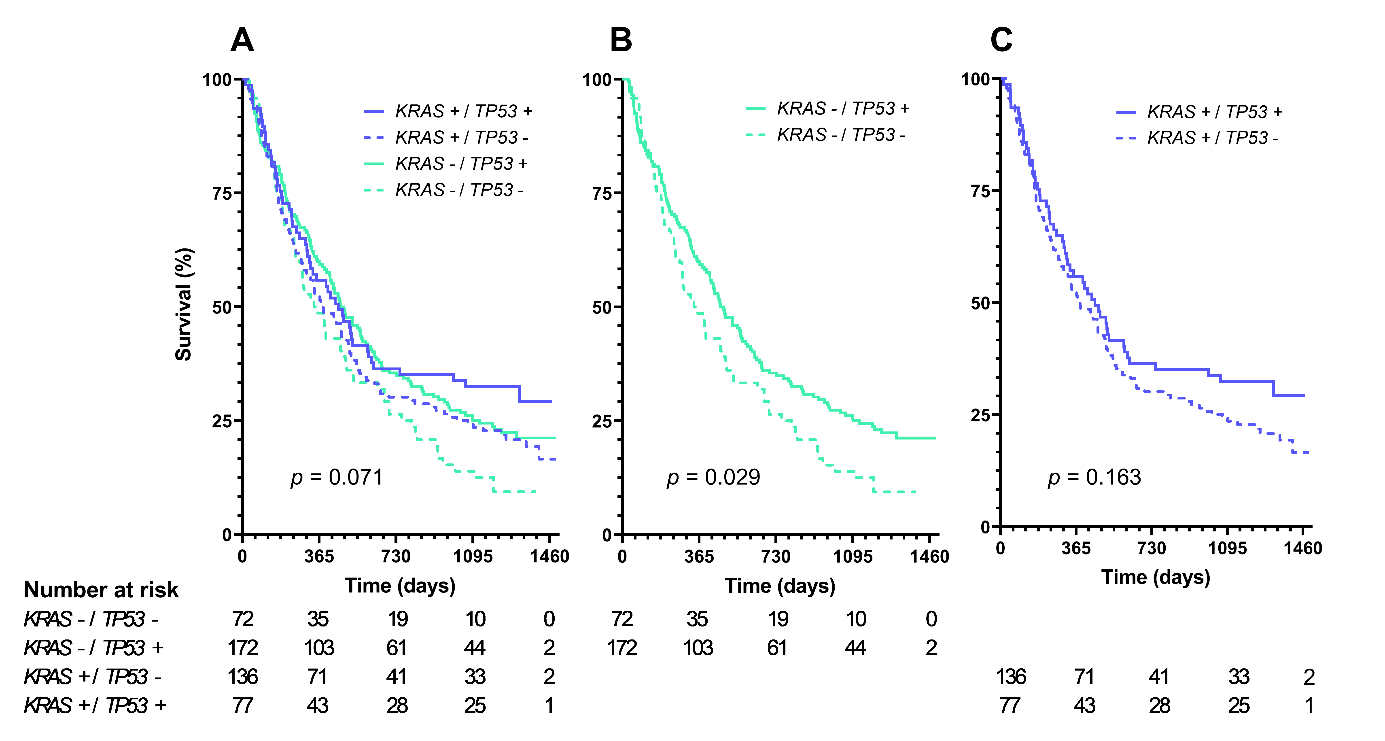
**Supplementary Figure S3**. Overall survival of metastatic NSCLC patients treated with first-line chemo-immunotherapy, stratified by *KRAS* and *TP53* mutational status: (A) all mutational subgroups, (B) *KRAS* wildtype only, and (C) mutated *KRAS* only. Presence of mutation in respective genes is indicated by ‘+’, lack of a (likely) pathogenic mutation is indicated by ‘-’. Data comprise patients diagnosed in 2019 and were retrieved from the Dutch National Cancer Registry (NCR) and the Dutch national pathology database (Palga).

**Supplementary Table S1.**

| **Supplementary Table S1.** Univariable and multivariable Cox regression analyses | | | |
| --- | --- | --- | --- |
| *Univariable Cox regression analysis* | | | |
| **Variable** | **OR** | **95% CI** | ***p*-value** |
| Age (per one year increase) | 1.01 | 1.00-1.02 | **0.008** |
| Male sex (compared to female sex) | 1.17 | 0.98-1.38 | 0.077 |
| Clinical stage of metastatic disease (compared to cM1a) |  |  | **0.001** |
| cM1b | 0.80 | 0.59-1.08 | 0.135 |
| cM1c | 1.25 | 1.00-1.56 | 0.050 |
| Chemo-immunotherapy (compared to mono-immunotherapy) | 1.23 | 1.03-1.48 | **0.024** |
| PD-L1 TPS (compared to ≥50%) |  |  | **<0.001** |
| 1-49% | 1.13 | 0.90-1.43 | 0.286 |
| <1% | 1.78 | 1.47-2.17 | **<0.001** |
| *KRAS*/*TP53* mutation profile (compared to *KRAS*mut/*TP53*mut) |  |  | **0.007** |
| *KRAS*wt/*TP53*wt | 1.67 | 1.24-2.25 | **<0.001** |
| *KRAS*wt/*TP53*mut | 1.25 | 0.98-1.60 | 0.073 |
| *KRAS*mut/*TP53*wt | 1.37 | 1.06-1.77 | **0.017** |
| *Multivariable Cox regression analysis* | | | |
| **Variable** | **OR** | **95% CI** | ***p*-value** |
| Age (per one year increase) | 1.01 | 1.00-1.02 | **0.009** |
| Male sex (compared to female sex) | 1.16 | 0.97-1.38 | 0.102 |
| Clinical stage of metastatic disease (compared to cM1a) |  |  | **0.001** |
| cM1b | 0.82 | 0.60-1.13 | 0.229 |
| cM1c | 1.29 | 1.03-1.61 | **0.028** |
| Chemo-immunotherapy (compared to mono-immunotherapy) | 0.76 | 0.54-1.08 | 0.122 |
| PD-L1 TPS (compared to ≥50%) |  |  | **<0.001** |
| 1-49% | 1.47 | 1.02-2.11 | **0.038** |
| <1% | 2.26 | 1.60-3.19 | **<0.001** |
| *KRAS*/*TP53* mutation profile (compared to *KRAS*mut/*TP53*mut) |  |  | 0.803 |
| *KRAS*wt/*TP53*wt | 1.08 | 0.78-1.49 | 0.661 |
| *KRAS*wt/*TP53*mut | 0.99 | 0.77-1.28 | 0.959 |
| *KRAS*mut/*TP53*wt | 1.10 | 0.84-1.43 | 0.503 |
